# *PTEN* hamartoma tumor syndrome in childhood and adolescence—a comprehensive review and presentation of the German pediatric guideline

**DOI:** 10.1186/s40348-022-00135-1

**Published:** 2022-02-21

**Authors:** Michaela Plamper, Bettina Gohlke, Joachim Woelfle

**Affiliations:** 1 Pediatric Endocrinology and Diabetology Division, Children’s Hospital, University of Bonn, Venusberg-Campus 1, 53127 Bonn, Germany; 2 Children’s and Adolescents Hospital, University of Erlangen, Erlangen, Germany

**Keywords:** PHTS, Guideline, Childhood, Adolescence, Diagnostic, Treatment, Cancer surveillance, Management

## Abstract

**Background:**

The *PTEN* hamartoma tumor syndrome (PHTS) encompasses several different syndromes, which are linked to an autosomal-dominant mutation of the tumor suppressor *PTEN* gene on chromosome 10. Loss of *PTEN* activity leads to an increased phosphorylation of different cell proteins, which may have an influence on growth, migration, and apoptosis. Excessive activity of the PI3K/AKT/mTOR pathway due to *PTEN* deficiency may lead to the development of benign and malignant tumors and overgrowth. Diagnosis of PHTS in childhood can be even more challenging than in adulthood because of a lack of well-defined diagnostic criteria. So far, there are no official recommendations for cancer surveillance in affected children and adolescents.

**Main body:**

All individuals with PHTS are at high risk for tumor development and thus might benefit from cancer surveillance strategies. In childhood, macrocephaly may be the only evident symptom, but developmental delay, behavioral problems, dermatological features (e.g., penile freckling), vascular anomalies, lipoma, or enlarged perivascular spaces in cerebral magnetic resonance imaging (cMRI) may help to establish the diagnosis. Regular psychomotor assessment and assistance in subjects with neurological impairment play an important role in the management of affected children. Already in early childhood, affected patients bear a high risk to develop thyroid pathologies. For that reason, monitoring of thyroid morphology and function should be established right after diagnosis. We present a detailed description of affected organ systems, tools for initiation of molecular diagnostic and screening recommendations for patients < 18 years of age.

**Conclusion:**

Affected families frequently experience a long way until the correct diagnosis for their child’s peculiarity is made. Even after diagnosis, it is not easy to find a physician who is familiar with this rare group of diseases. Because of a still-limited database, it is not easy to establish evidence-based (cancer) surveillance recommendations. The presented screening recommendation should thus be revised regularly according to the current state of knowledge.

## Background

The *PTEN* hamartoma tumor syndrome (PHTS) compromises several different syndromes, which are linked to an autosomal-dominant mutation of the tumor suppressor gene *PTEN* on chromosome 10. Loss of *PTEN* activity leads to an increased phosphorylation of different cell proteins, which may have an influence on growth, migration, and apoptosis. Excessive activity of the PI3K/AKT/mTOR pathway may lead to the development of benign and malignant tumors and overgrowth. Cowden Syndrome (CS) and Bannayan–Riley–Ruvalcaba syndrome (BRRS) are associated to *PTEN* gene mutations, but also Proteus/Proteus-like syndrome, autism spectrum disorders with macrocephaly, Lhermitte–Duclos syndrome, and juvenile polyposis of infancy have been linked to *PTEN* gene mutations. All individuals with PHTS are at high risk for tumor development and benefit from cancer surveillance strategies, because in general, tumor detection in an early stage may lead to lower mortality rates and decreased risk for complications. There is no clear evidence for differences in the incidence of different types of cancer in the different *PTEN*-attributed syndromes or a distinct genotype–phenotype correlation. Therefore, at the current state of knowledge, we are not able to differentiate screening recommendations for the different syndromes. The most frequent affected organs are the breast, endometrium, and thyroid. Thyroid cancer has been reported already in very young children [1–3]. Most patients exhibit an increased head circumference already early in life (more than + 2 SDS above the age and population–related mean or > 97th percentile) [4–8]. Our own data showed that all patients exhibited a head circumference > 97th percentile by the age of 2 years. Most male patients already showed a macrocephaly at birth, and the extend was more pronounced than in female patients [8]. Developmental delay, especially a delay in motor development is described frequently without a clear genotype–phenotype correlation. PHTS is a rare disease with a prevalence of 1:200.000 to 1:250.000 [9, 10]. The National Comprehensive Cancer Network (NCCN) published guidelines for diagnosis and treatment of adult PHTS patients [11]. However, official pediatric guidelines are lacking. Children frequently do not fulfill the known diagnostic criteria for adult patients. Therefore, diagnosis of PHTS in childhood may be even more challenging than in adulthood. Furthermore, cancer surveillance strategies should be different, since the risk of tumor development in childhood differs from adult patients. Mostly, malignant tumors are reported in adults. In children, there are only case reports of malignant tumors, except for the thyroid, which is frequently affected. With this article, we would like to increase knowledge of this rare disease, in order to facilitate an earlier diagnosis of affected subjects. Supported by the German Society for Pediatric Endocrinology and Diabetology (DGKED) and in cooperation with several other societies (German Society for Human Genetics (GfH), German Society of Pediatrics (DGKJ) German Society for Pediatric Oncology and Hematology (GPOH), German Society for Neuropediatrics (GNPI), German Society for Pediatric Gastroenterology and Nutrition (GPGE), and the German Patient Support Group (CoBald)), we developed a pediatric guideline for the diagnosis and management of PHTS in childhood and adolescence, in order to aid physicians in the diagnosis, surveillance, and management of their patients. For this guideline, we performed a nonsystematic search in https://pubmed.ncbi.nlm.nih.gov including, but not limited to, the following key words: “PTEN Hamartoma Tumor Syndrome AND children”, “PHTS AND Children AND Thyroid”, “PTEN AND Children AND Thyroid”, “PHTS AND Guideline”, “Macrocephaly AND PTEN”. This search was extended by publications cited by some of the keyword-driven publications, finally updated in July 2021.

### Syndromes and phenotypes which are associated with *PTEN* hamartoma tumor syndrome (PHTS)

#### Cowden syndrome and Bannayan–Riley–Ruvalcaba syndrome (BRRS)

Cowden syndrome is the most commonly known syndrome associated with a germline mutation of the tumor suppressor gene *PTEN*. BRRS and Cowden syndrome can be seen as one condition with variable expression and age-related penetrance [4, 12]. Within this perspective, BRRS describes the typical manifestation of the disease in childhood, whereas Cowden syndrome describes the clinical manifestation more evident in adulthood. There is a smooth transition between both entities. Typical clinical manifestations for both syndromes are an increased head circumference above the 97th percentile and a variety of hamartomata, such as hemangioma, lipoma, and gastrointestinal polyps. The latter may cause intussusception or rectal bleeding [4–8]. PHTS patients are at high risk to develop benign and malignant tumors.

#### Proteus syndrome/Proteus-like syndrome

Proteus syndrome is a very rare condition and quite variable in its phenotype. Overgrowth of different body parts like skin, bone, muscles, fat tissue, and blood or lymph vessels is frequently observed. The risk for tumor development is significantly increased. It is usually associated with a mutation in the *AKT-1*-gene. In rare cases, mutations in the *PTEN* gene have also been described. Except for the macrocephaly, the clinical presentation of children with Proteus syndrome differs relevantly from patients with CS or BRRS. Their specific treatment is not part of this review.

#### Autism spectrum disorder with macrocephaly

In patients with autism spectrum disorder in combination with macrocephaly, a *PTEN* gene mutation has been detected in 10 to 27% [13–16]. The currently available data suggest that the likelihood of identifying *PTEN* mutations is higher in individuals with autism spectrum disorder with more pronounced macrocephaly [15, 17].

#### Lhermitte–Duclos syndrome

Lhermitte–Duclos syndrome is a variant of CS and is characterized by slowly growing hamartomatous tumors of the cerebellum (cerebellar dysplastic gangliocytoma) and occurs typically in adults. Associated symptoms depend on the tumor size and include ataxia, elevated intracranial pressure, and seizures.

#### Juvenile polyposis of infancy

Juvenile polyposis of infancy is a very rare condition and is caused by deletions of the *BMPR1A* and *PTEN* genes. Patients suffer from gastrointestinal bleeding, diarrhea, and a severe protein-losing enteropathy. Further clinical manifestations might resemble BRRS [16].

### Clinical phenotype and respective monitoring recommendations of *PTEN* hamartoma tumor syndrome in children and adults

Recommendations for cancer surveillance of adults with PHTS were previously published by NCCN and others [11, 18, 19]. However, children and adolescents with PHTS were not or inadequately considered in those recommendations. Therefore, we propose a specific monitoring regime for children and adolescents. After diagnosis of a *PTEN* gene mutation in children, we recommend timely genetic counselling including the opportunity for an analysis regarding a *PTEN* gene mutation in relatives (especially parents and siblings). Affected families need to be informed about the variability of the clinical spectrum and potential complications of this rare disease. If possible, the explaining clinician should have expertise in this field. All patients with PTEN gene mutations should have an annual comprehensive physical examination.

### Central nervous system

#### Macrocephaly

As mentioned above most patients with PHTS exhibit a marked macrocephaly early in life (up to 2 years of life) with a head circumference standard deviation score of + 3 SDS and more. Therefore, head circumference, but neither height nor weight development, might be useful to establish an earlier diagnosis of PHTS [8].

#### Development, intelligence, and imaging in cerebral MRI

Frequently, children with PHTS demonstrate a variable degree of developmental delay. Most patients show a delay in motor development. A fraction of patients presents with muscle hypotonia and proximal myopathy, which at the time might be the leading clinical problem. Intellectual disabilities may be present, but with a high variability [16] from severe cases to subjects with normal intelligence. In our cohort and in others [20, 21], the majority of patients had a mean intelligence quotient (IQ) within the normal range. The prevalence of autism spectrum disorders in patients with PHTS is estimated around 22% [22, 23] and therefore higher than in the general population with around 1% [24].

**Fig. 1:**
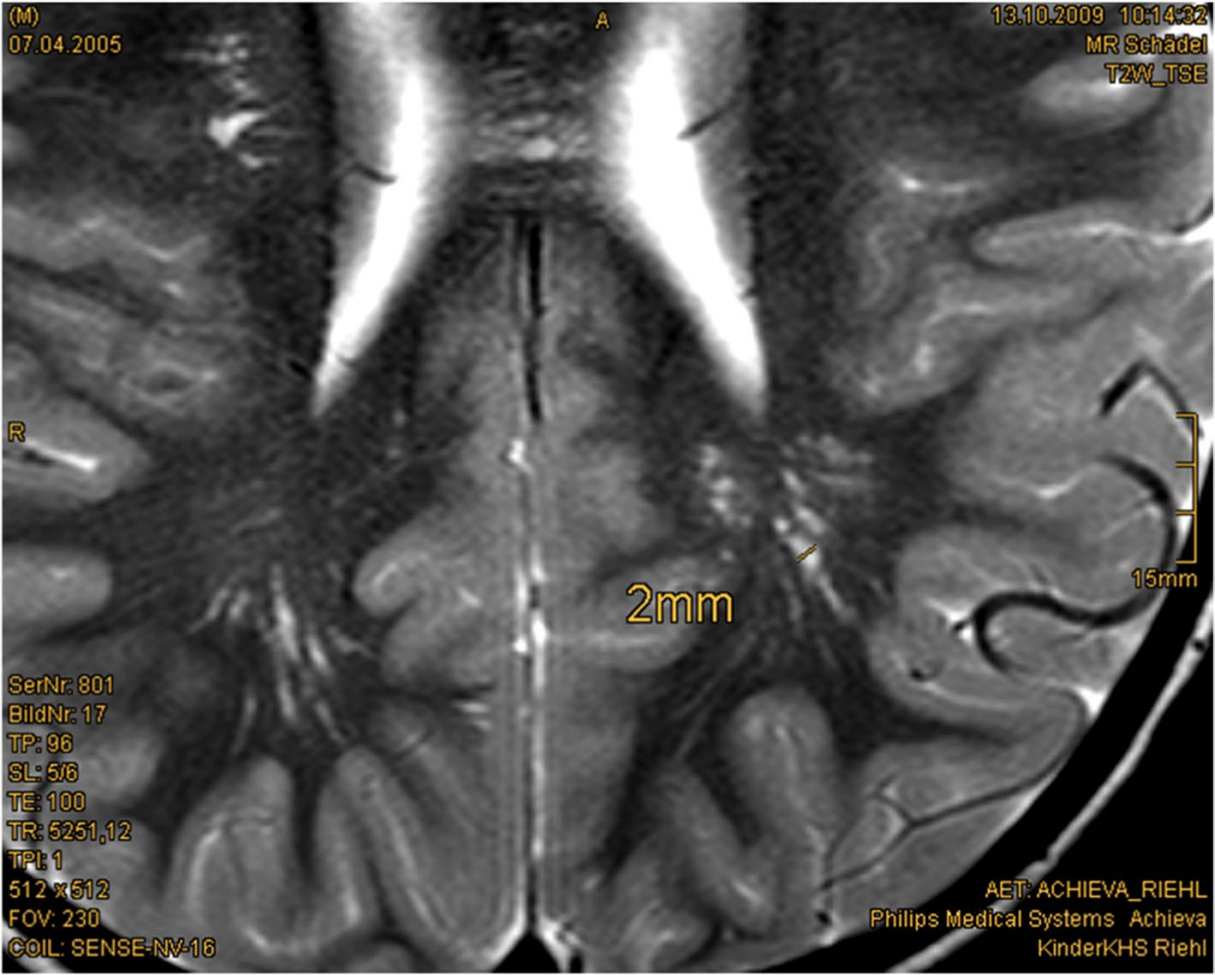
Enlarged perivascular spaced (EPVS) in cerebral MRI: 4.5-year-old boy, T2w-image, EPVS 2 mm diameter [Courtesy Kinderkrankenhaus Kliniken der Stadt Köln]

Most children with PHTS receive a cerebral MRI as part of the diagnostic workup of macrocephaly. A high percentage shows enlarged perivascular spaces (EPVS) and white matter abnormalities. Therefore, detecting these abnormalities in cerebral magnetic resonance imaging (cMRI) might also help in establishing an earlier diagnosis in PHTS patients [20] (Fig. 1). In childhood, psychomotor assessment and, if necessary, further neurological diagnostic and treatment play a central role in the management of patients with PHTS. Muscle hypotonia might be treated with physiotherapy or supporting medical devices. In case of severe neurological symptoms or recurrent headaches, a cerebral MRI should be performed. In adults, cerebellar dysplastic gangliocytoma (Lhermitte–Duclos syndrome) should be excluded. Vascular malformations could be located anywhere and cause diverse symptoms. To the best of our knowledge, there is no proved genotype–phenotype correlation for the extent of neurological symptoms.

#### Skin

**Fig. 2:**
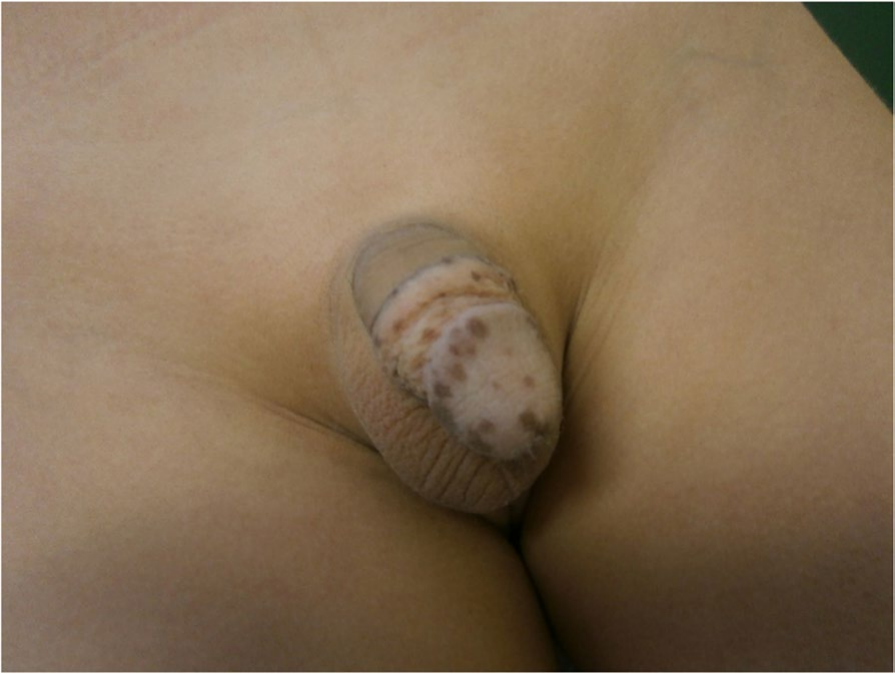
Penile freckling

Typical skin manifestations in childhood are hamartomata such as lipoma and hemangioma. In males with a *PTEN* gene mutation, penile freckling could be a useful diagnostic feature, since it is described in about half of all male cases [18, 20]. Penile freckling (Fig. 2) may not be evident in infants, but usually develops until mid-childhood. Later in life, mostly in the second or third decade, nearly all patients exhibit typical mucocutaneus symptoms, for example trichilemmoma, oral mucosa papillomatosis, palmoplantar keratosis, or hyperplasia of the gingiva [18]. The risk for melanoma seems to be elevated in patients with PHTS [18]. Cumulative lifetime risk for melanoma is reported to be around 6% [19] with the youngest patients being reported to be 3 [19] and 6 [18] years old. Even though there are only single reports of melanoma in children with PHTS, we recommend yearly dermatological examinations and advice to take care of UV protection.

### Benign and malignant tumors

Subjects with PTEN gene mutation harbour an increased risk for tumor development of thyroid malignancies occurring already in young children. Riegert-Johnson [25] estimated cumulative lifetime (age 70 years) risks of 89% for any cancer diagnosis.

#### Breast

**Fig. 3:**
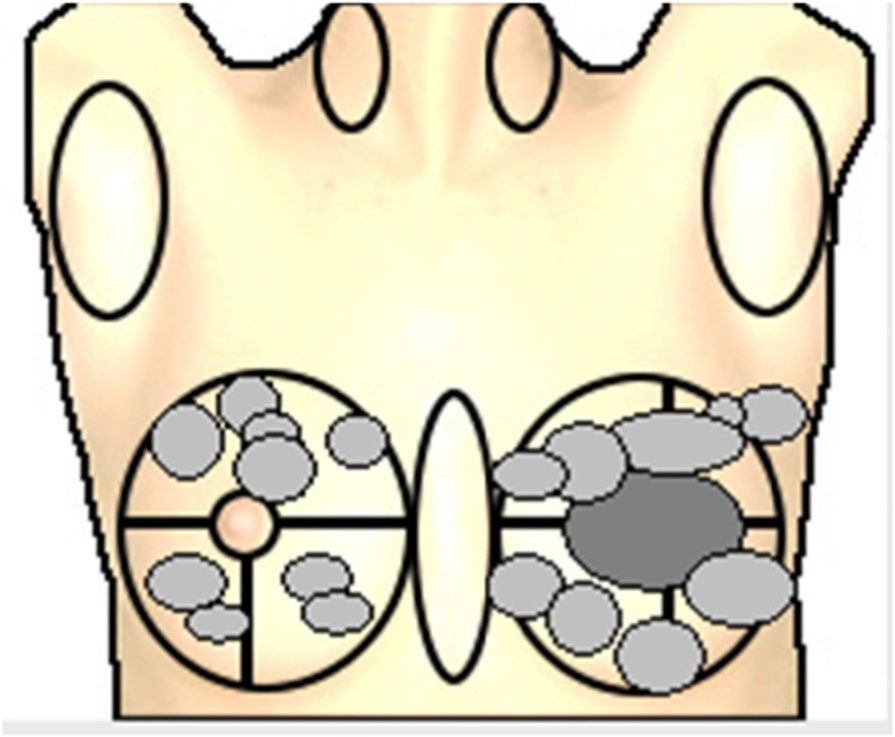
Fibroadenoma distribution in both mammae of a 14-year-old girl

Almost all women with *PTEN* gene mutation have an involvement of the breast [26], including benign and malignant tumors. Already adolescents may develop multiple benign tumors of remarkable size, for example fibroadenoma, tubular adenoma, or atypical ductal hyperplasia (Fig. 3). To the best of our knowledge, breast cancer has been described only in adult women, even though there are case reports of very young women (22 years) with PHTS suffering from breast cancer [26]. Similar to women who carry BRCA gene mutations, women with PHTS tend to develop breast cancer in younger years. Mean age at diagnosis is 36 to 46 years [9]. Riegert-Johnson et al. [25] report a cumulative lifetime risk of breast cancer in women with PHTS of 81%. Other authors estimate the risk between 25 to 85% [18, 22, 27]. Single cases of breast cancer have also been reported in men [28–30].

We recommend, that systematic breast awareness of females with a *PTEN* gene mutation should start at the age of 18 years monthly. Clinical examination is recommended at the age of 25 years (or 5–10 years before youngest age of cancer diagnosis in the family) twice per year. MRI of the breast and mammography are recommended to start latest at the age of 35 years (or 5–10 years before youngest age of cancer diagnosis in the family).

#### Thyroid

Up to 75% of all patients demonstrate thyroid pathologies such as nodules, goiter, and autoimmune thyroid disease [1]. The cumulative lifetime risk of patients with *PTEN* gene mutations to develop thyroid carcinoma is estimated between 21 and 38% [16, 18, 22, 25, 31]. In most cases, patients develop follicular carcinoma, rarely papillary thyroid cancer. Single cases of medullary carcinoma have been described [31]. The development of thyroid pathologies, including thyroid cancer might occur already early in life [1–3, 19], with the youngest reported patient with a thyroid carcinoma being 6 years old [1]. Pediatricians should be aware of the early thyroid involvement and possible thyroid cancer development in PHTS patients. We found no evidence for an improved outcome in children with PHTS and an early detection of thyroid carcinoma. But some evidence could be found in non PHTS patients, especially for adults. Detection in an early stage leads to a lower mortality rate (high level of evidence for adults, very low level of evidence for children) and a decreased risk of hypoparathyroidism (high level of evidence for adults) [32], and it was found to lead to lower recurrence rates (moderate to low level of evidence for adults; very low level of evidence for children).

Therefore, we recommend annual ultrasound screening of the thyroid, independent of the age at diagnosis. We propose an annual examination interval, which should be adapted in case of any suspicious results [1]. Beginning and interval of surveillance are a subject of discussions. Jonker et al. [33] analyzed five cohort studies where the incidence for differentiated thyroid carcinoma ranged between 4 and 12%, and recommended to start screening for differentiated thyroid carcinoma, when the risk exceed 5%. This is in concordance to other childhood tumors with an excellent prognosis. In their analysis, most cases were diagnosed between the ages of 10 and 14 years, so that they recommend surveillance from the age of 10 years onwards, since at that age, the incidence of thyroid carcinoma seems to reach 5%. Smith et al. [34] performed a retrospective cohort study of 64 children with PHTS. Clinically significant thyroid nodules (≥ 10 mm diameter) developed in 44% of 50 patients who underwent thyroid ultrasound at a median age of 13.3 years. Nodules were rare prior to the age of 7 years. Prevalence was increasing around the age of puberty with an earlier appearance in girls. Thyroid cancer was diagnosed in 2 patients (4%). Their findings indicated, that children without or with only small nodules (< 5 mm) were unlikely to develop significant nodules ≥ 10 mm within 2 years, whereas nodules ≥ 5 mm showed a likelihood of 20% to grow ≥ 10 mm within 1 year. Both groups [33, 34] elaborate that early screening might lead to overdiagnosis and anxiety of families. On the other hand, another group [35] with a small case series reported that nearly half of their subjects, who had nodular thyroid disease were younger than 7 years of age. Based on these results [33–35], the interval of surveillance may be prolonged two biannual or every 3 years in young children (< 7 years of age) if they do not have proof of nodules in ultrasound screening. If there is a nodule with suspicious ultrasound findings (e.g., size > 10 mm, central hyperperfusion, irregular margin, microcalcifications, quickly growing size), further diagnosis of dignity is indicated. We recommend fine-needle biopsy (FNB), where it is both appropriate and possible. However, we do see some limitations for FNB. Most pediatric patients, especially those who may have behavioral specifics like autism would frequently need general anesthesia to undergo this procedure. Fine-needle biopsy may not be sufficient to exclude malignancy in cases of more than one suspicious lesion, which is common in patients with PHTS. It is not possible to distinguish a benign follicular adenoma from a follicular carcinoma by cytological analysis, but most PHTS patients exhibit follicular lesions [36]. Because of multicentricity and increased risk of recurrence or progression to carcinoma [2, 11, 37, 38], we agree with other groups that total thyroidectomy should be recommended when a surgical intervention is indicated. Surgical intervention should be performed by an endocrine surgeon with experience in the treatment of children.

#### Urogenital system (endometrium, gonads, renal system)

The lifetime risk to develop endometrial carcinoma in women is reported to be 19–28% [19, 25] compared with the general population with 2.1% with a mean age of 69 years [31]. Except for one 16-year-old girl with granulosa cell tumor, gonadal tumors were not described in PHTS [3]. Testicular lipomatosis is described in adult males with *PTEN* gene mutation. We recommend testicular ultrasound at the beginning of puberty around the age of 10 years. The cumulative lifetime risk (age 70 years) for renal cancer is elevated and appraised to be around 15% (*CI* = 6%, 32%) [25]. As the youngest patient with a PHTS associated renal cell carcinoma was diagnosed at the age of 11 years [3], we recommend abdominal ultrasound once every year, starting with diagnosis, even though most cases of renal tumors were reported in adult subjects.

#### Gastrointestinal manifestations

In 95% of affected subjects, the gastrointestinal tract is part of the phenotypical spectrum [39–44]. This encompasses hamartomatous, inflammatory, adenomatous, ganglioneuromatous, hyperplastic, and juvenile polyps which may be located in all parts of the gastrointestinal tract with a focus on the rectum and sigmoid. Dissemination and severity may differ between cases. A typical clinical manifestation is perianal bleeding. The lifetime risk to develop colon carcinoma is estimated from 9 [22] to 16% [25]. Age at diagnosis of colon carcinoma is mostly around 50 years of age [39]. There are no descriptions of colon carcinoma in childhood. Acanthosis of esophagus [5, 42] and eosinophilic esophagitis [43] may be further gastrointestinal pathologies in young patients with PHTS. Even though hamartomatous polyps are found early in childhood, the majority of children are not symptomatic. In contrast, patients with a deletion of the *BMPR1A* and *PTEN* gene show a severe clinical picture of juvenile polyposis of infancy and need early gastrointestinal diagnostics and treatment. The American guideline for hereditary gastrointestinal cancer syndromes [41] recommends gastroscopy and colonoscopy for patients with PHTS starting at the age of 15 years every 2 years. Other authors point out different recommendations starting at the age of 35 years [16, 45], because colorectal tumors have been mostly diagnosed at the end of the fourth decade of life [22]. We recommend to differentiate whether patients show symptoms or not. In patients without any complaints, endoscopy to detect tumor development seems to be sufficient at the age of 35 years. In contrast, patients with symptoms like hematochesis, severe constipation, diarrhea, or recurrent and severe abdominal pain should receive early diagnostic intervention. Because rectal bleeding and the development of anemia could be symptoms of polyps, we recommend to screen for bleeding anemia.

#### Lipomatosis

**Fig. 4:**
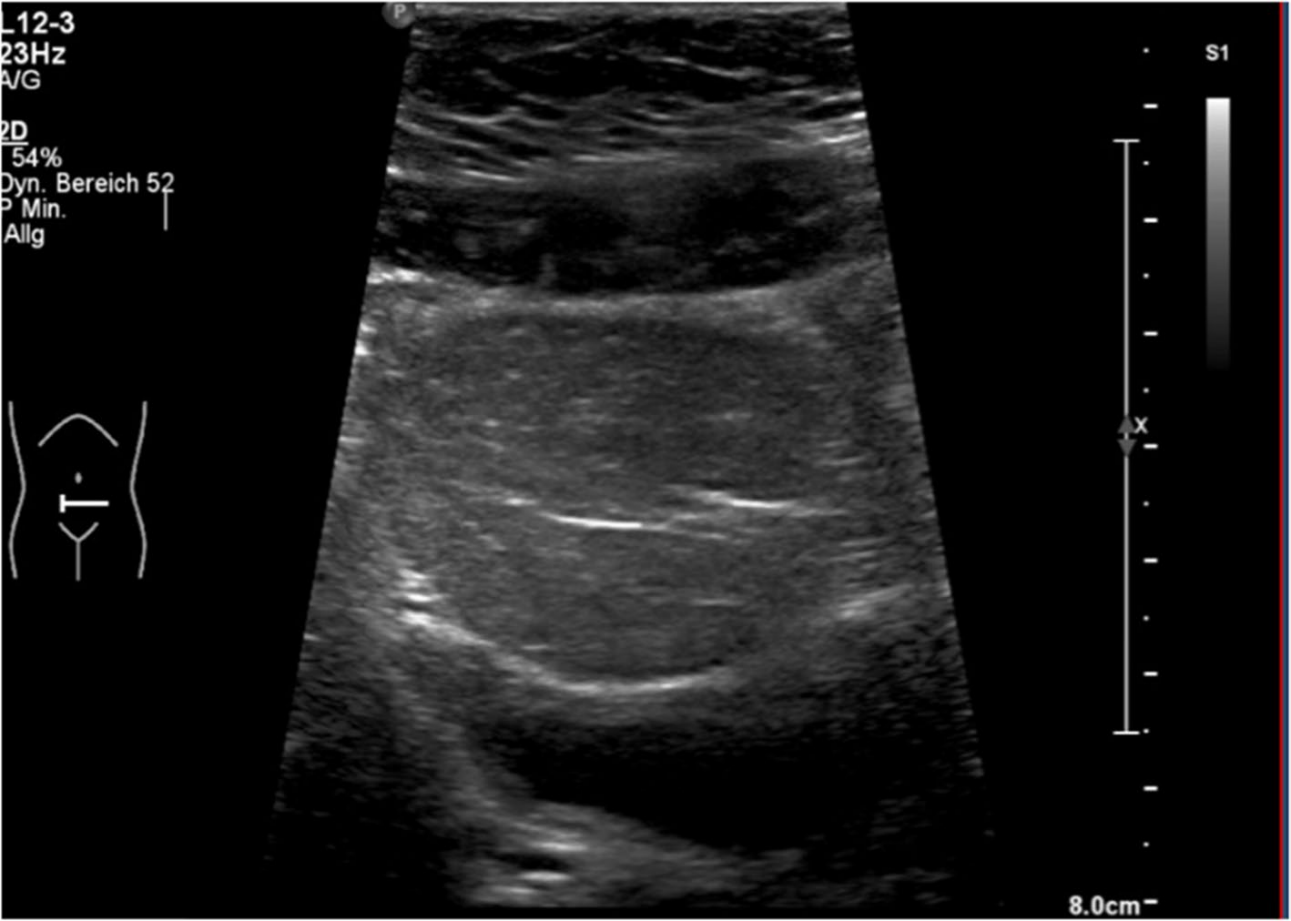
Intraabdominal lipoma compromising urinary bladder in a 10-year-old girl

A lipoma may develop a remarkable size and may therefore have space compromising effects, depending on their localization (Fig. 4). Size and growth might be monitored by ultrasound or MRI scan. As long as there are no space-compromising effects or cosmetic impairments, lipomas do not need an intervention.

#### Vascular anomalies

Regulation of angiogenesis is one function of the *PTEN* gene. Loss of *PTEN* gene function could therefore be associated with vascular anomalies. Vascular anomalies/malformations can be localized at any part of the body. Prevalence of vascular anomalies in PHTS is still unclear, but some reports estimated the presence of these in at least one third of the affected subjects [22, 44]. Tan et al. [46] present a collective of 26 pediatric patients of whom 14 patients presented vascular anomalies. The age at first vascular symptom, defined to the age when the vascular anomaly was first noticed by either the patient or doctor, ranged from birth (three patients) to 16 years of age. Vascular anomalies usually present as cutaneous discoloration, swelling, or pain [22, 46]. Physicians should be aware of possible symptoms of vascular anomalies in physical examination and ask the patients and parents, if they have any symptoms of skin discoloration, swelling, or pain. Dural [22], intracranial developmental venous anomalies (DVAs) [46] and large visceral [47], vertebral [48], and intestinal arteriovenous malformations (AVMs) [44, 49] have been reported. Intestinal arteriovenous malformations may lead to extensive blood loss and severe anemia [44, 49]; vertebral AVMs may lead to destruction of the vertebral body [48]. Takaya et al. [50] described the management of a patient with a clinical diagnosis of CS who presented with multiple large AVMs at the age of 30 years. The lesions caused high-output cardiac failure and subsequent death of the patient. Clinicians should be aware of the risk of hemodynamically significant AVMs in PHTS [47]. For more detailed information of the anomalies, imaging diagnostics should be added (for example sonographic images, angio-MRI).

#### Immunology

Chen et al. [51] found autoimmunity and peripheral lymphoid hyperplasia in 43% of 79 patients with PHTS. Lymphoid hyperplasia may include gastrointestinal lymphoid hyperplasia, extensive hyperplastic tonsils, and thymus hyperplasia; autoimmunity may include autoimmune lymphocytic thyroid disease, autoimmune hemolytic anemia, and colitis [52]. Immune dysregulation in patients with PHTS include lymphopenia, CD41 T-cell reduction, and changes in T- and B-cell subsets [51]. Although total CD41FOXP31 Treg cell numbers are reduced, frequencies are maintained in the blood and intestine. Despite pathogenic *PTEN gene* mutations, the FOXP31 T cells are phenotypically normal. Assembly of the *PTEN*-PHLPP phosphatase network allows coordinated phosphatase activities at the site of T-cell receptor activation, which is important for limiting PI3K hyperactivation in Treg cells despite *PTEN* haploinsufficiency [51]. Reduced activity of *PTEN* affects homeostasis of human germinal center B cells by increasing PI3K-AKT signaling via mammalian target of rapamycin as well as antiapoptotic signals [52]. Everyday, clinical relevance of the immunological changes in PHTS is still unclear. Therefore, at the current time, we cannot give substantial clinical recommendations.

#### Metabolism and obesity

*PTEN* haploinsufficiency seems to be a monogenic cause of profound constitutive insulin sensitization that is apparently obesogenic [53]. The patients’ insulin sensitivity could be explained by the presence of enhanced insulin signaling through the PI3K-AKT pathway, as evidenced by increased AKT phosphorylations [53]. In this context, there are several case reports of children with *PTEN* gene mutations and recurrent hypoglycemia [54–56]. In our own cohort of children [8] with *PTEN* gene mutations, the majority of patients had a BMI-SDS above the 50th percentile. The percentage of overweight and obesity in the younger age groups was elevated compared with the German reference data [57].

### Diagnostic criteria of PHTS and indications for genetic analyses

**Fig. 5:**
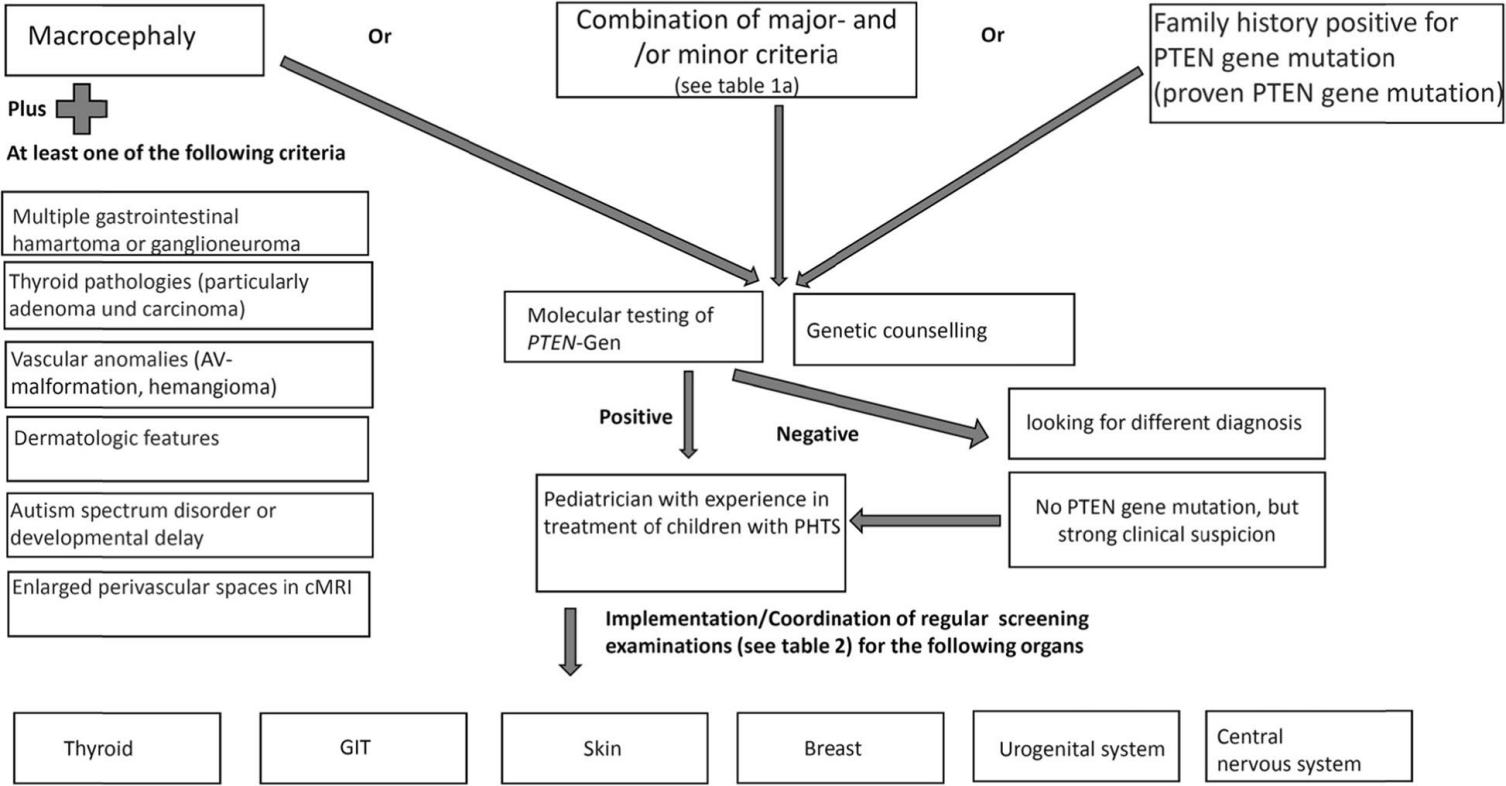
Flow process chart: indication for genetic diagnostics of *PTEN* gene mutation and proceedings after positive mutation detection

The National Comprehensive Cancer Network (NCCN) [11] and others [58] published recommendations for diagnostic criteria of PHTS, distinguishing between major criteria and minor diagnostic criteria. In case, that at least three major criteria or two major and three minor criteria are fulfilled or if there is a familiar case and two major or one major and two minor criteria are fulfilled, molecular testing for *PTEN* gene mutation should be initiated. Tan et al. [22] pointed out that significant differences in the clinical phenotype of children and adults with *PTEN* gene mutation exist, with all children in the reported study exhibiting macrocephaly. The majority showed developmental delay or some kind of autism spectrum disorder. Because children show different or less symptoms compared with adults, Tan et al. proposed specific diagnostic criteria for children. Comparably, in the German pediatric guideline, we propose a modified diagnostic algorithm for children and adolescents with *PTEN* gene mutations (see Tables 1,2, and 3; Fig. 5). This diagnostic algorithm addresses all subspecialties who care for children, with focus on general pediatricians, pediatric neurologists, radiologists, pediatric endocrinologists, and geneticists. In case of doubt, the patients should be presented to a clinical geneticist.

**Table 1:** Major and minor criteria for indicating molecular testing of the *PTEN* gene mutation in children and adolescents (modified from [20, 22, 59])

Major criteria	Minor criteria
Macrocephaly	Autism spectrum disease
Family history positive for PTEN gene mutation	Mental retardation (i.e., *IQ* < 75)
Dermatological findings like - Trichilemmoma - Oral papilloma - Penile freckling	cMRI findings like - Enlarged perivascular spaces - White matter abnormalities
Vascular anomalies/malformations	Lipoma/lipomatosis
Multiple gastrointestinal hamartoma or ganglioneuroma	Esophageal acanthosis
Thyroid adenoma and thyroid carcinoma	Other thyroid lesions (e.g., multinodular goiter, autoimmune thyroid disease)
Breast cancer	Testicular lipomatosis
Endometrial cancer	Renal carcinoma

**Table 2:** Clinical criteria for molecular analysis of the *PTEN* gene in children and adolescents (modified from Tan et al.)

Molecular analysis for PTEN gene mutation, if:	Macrocephaly plus at least one of the following symptoms	No macrocephaly, no suggestive family history, but	Family history positive for ***PTEN*** gene mutation
	Autism spectrum disorder or developmental delay	Two major criteria	Molecular analysis for *PTEN* gene mutation, if one parent is carrier of a mutation.
	Dermatologic features: penile freckling, lipoma, trichilemmomas, oral papillomas, hemangioma	One major criterion plus two minor criteria	
	Multiple gastrointestinal hamartomata or ganglioneuromata		
	Vascular anomalies	Three minor criteria	
	Thyroid pathologies (particularly adenoma and carcinoma)		
	Enlarged perivascular spaces in cMRI

**Table 3:** Possibly affected organs, possible pathologies, and screening recommendations for children and adolescents with PHTS (< 18 years of age)

Organ	Possible pathologies	Screening recommendation	Screening frequency
Thyroid	Adenoma, follicular and papillar thyroid carcinoma, goiter, autoimmune thyroid disease	Thyroid ultrasound (starting with diagnosis)	**At least annual** (in children < 7 years of age and without nodules: every 2–3 years)
GIT	Hamartomatous polyps, esophageal acanthosis, carcinoma	Gastro-/colonoscopy in patients without symptoms: individual decision in childhood Gastro-/colonoscopy in patients with symptoms (e.g., hematochesis, severe constipation, diarrhea, recurrent and severe abdominal pain): diagnostic should be planned promptly. Regular gastro-/colonoscopy starting at age 35.	Depending on diagnostic findings and symptoms Depending on diagnostic findings and symptoms Every 5 years
Female breast	Benign and malignant tumors of the breast	Breast awareness beginning at 18 years of age Clinical breast examination beginning at 25 years of age (or 5–10 years before youngest age of cancer diagnosis in family) Breast MRI and mammography at age 30–35 in women (or 5–10 years before youngest age of cancer diagnosis in family)	Every month 1–2 times/year Annual
Skin	Benign and malignant tumors	Dermatological exam	Annual
Urogenital system	Renal carcinoma (in adults) Testicular lipomatosis/ endometrial cancer (in adults)	Abdominal ultrasound starting with diagnosis Ultrasound of testes/uterus and ovaries beginning at 10 years of age	Annual Every 1–2 years
CNS	Developmental delay, white matter abnormality, enlarged perivascular spaces, autism spectrum disorder, cerebellar dysplastic gangliocytoma in adults, meningioma (rare cases)	Psychomotor assessment cMRI in the presence of neurological signs and symptoms	Depending on imaging/neurological phenotype

### Therapy

Treatment of benign and malignant tumors in patients with *PTEN* gene mutation does not differ from treatment strategies in other patients.

#### Medical treatment

*PTEN* gene encodes a phosphatase, which regulates PI3K-AKT-mTor signaling. As a result of the mutation, excessive activity of this pathway leads to overgrowth and tumor development. Inhibition of mTOR signaling is a promising target for a possible medical treatment. Single case reports have described the successful use of the mTOR inhibitor sirolimus in patients with segmental overgrowth and *PTEN* germline mutation [60–63]. There are ongoing investigations on sirolimus treatment in segmental overgrowth syndromes that also include children. Because of the still-limited state of knowledge at the time, mTOR inhibitors should only be used in clinical studies or on a carefully proofed case-by-case basis.

#### Surgery

Surgical treatment of patients with PHTS does not differ from respective treatment of other patients. In case of thyroid nodules where malignancy could not be excluded, we recommend total thyroidectomy, because of the risk of multicentricity and recurrence in patients with PHTS. Surgical intervention should be performed by an endocrine surgeon with experience in the treatment of children.

#### Psychomotor development and psychosocial and pedagogical aspects

Despite the possible tumor development and its treatment, patients and families often face numerous additional issues in children and adolescents with PHTS, that are quite or even more challenging. Affected children might exhibit variable degree of developmental delay, that might require treatment (e.g., by physiotherapy or ergotherapy). Some patients need supporting medical devices due to muscle hypotonia. Autism spectrum disorders and behavioral problems can play a major role in some patients. Intellectual abilities are very variable, and some patients might face problems in daily school life. For these reasons, neurological, physiotherapeutic, ergotherapeutic, and psychological treatments might be needed. If the child shows aspects on autism spectrum disorders, it is advisable to consult an expert for autism. To realize those problems and to possibly establish professional therapies, we recommend to ask for the psychological burden of patients and family at the annual comprehensive physical examination.

### Recommendations for children and adolescents with PHTS

In brief, recommendations are as follow:

1. Genetic counselling after diagnosis
2. Annual comprehensive physical examination
3. Psychomotor assessment in children and adolescents
4. Ultrasound examination of the thyroid right after diagnosis
  Annual examinations and interval of thyroid ultrasound, which has to be adapted in case of any suspicious result. Children < 7 years of age without proof of nodules in ultrasound screening may have a longer screening interval (2–3 years).
  We recommend a low threshold for early surgical intervention (preferably total thyroidectomy).
5. Awareness of possible rectal bleeding and anemia. Early diagnostic intervention in patients with gastrointestinal symptoms like hematochesis, severe constipation, diarrhea, and recurrent and severe abdominal pain. In asymptomatic patients, we recommend gastro- and colonoscopy by the age of 35 years for cancer surveillance at least every 5 years.
6. Breast awareness should start latest by the age of 18 years, clinical breast examinations by the age of 25 years, and imaging (breast MRI/mammography) by the age of 35 years or 5–10 years before youngest cancer diagnosis in family.
7. Yearly dermatological examination and adequate skin protection
8. Yearly abdominal ultrasound. Testicular/uterus and ovarian ultrasound starting around the beginning of puberty (with 10 years of age)
9. Performance of cMRI in the presence of neurological symptoms or signs
10. Medical treatment with mTOR inhibitors should only be used in clinical studies or on a carefully evaluated case-by-case basis
11. Surgical treatment does not differ from other persons
12. Surveillance of psychological burden of patients and families

## Conclusion

The *PTEN* hamartoma tumor syndrome is rare, and therefore, diagnosis is often delayed. Families of affected children frequently encounter problems to find medical staff who is familiar with symptoms, diagnosis, and treatment of children with PHTS. Parents often have a high burden concerning psychomotor development, behavioral problems, autism spectrum disorders, and the known elevated tumor risks of their child. There are only limited data of tumor prevalence in childhood, making it difficult to find an adequate balance between too much or too little and too early and too late cancer surveillance. Because of a still-limited database, it is not easy to establish evidence-based (cancer) surveillance recommendations. Most recommendations rely on expert opinions. The recommendations of this article have to be revised regularly on the current state of knowledge.

## Data Availability

Not applicable

## Abbreviations

PHTS: *PTEN* hamartoma tumor syndrome
cMRI: Cerebral magnetic resonance imaging
CNS: Central nervous system
CS: Cowden syndrome
BRRS: Bannayan–Riley–Ruvalcaba syndrome
DGKED: German Society for Pediatric Endocrinology and Diabetology
GfH: German Society for Human Genetics
DGKJ: German Society of Pediatrics
GPOH: German Society for Pediatric Gastroenterology and Nutrition
GNPI: German Society for Neuropediatrics
GPOH: German Society for Pediatric Oncology and Hematology
CoBald: German Patient Support Group
GIT: Gastrointenstinal tract
IQ: Intelligence quotient
EPVS: Enlarged perivascular spaces
FNB: Fine-needle biopsy
e.g.: For example
DVAs: Developmental venous anomalies
AVMs: Arteriovenous malformations
NCCN: National Comprehensive Cancer Network
